# Primary cardiac T-cell lymphoma presenting as ST-elevation myocardial infarction with fatal ventricular arrhythmias: a case report

**DOI:** 10.1093/ehjcr/ytag086

**Published:** 2026-01-31

**Authors:** Alaa Jamal Alobaidli, Muhammad Azam Shah, Abdullah Alkhodair, Halia Zain Alshehri, Mohammed Alhumaid

**Affiliations:** Department of Adult Cardiology, King Fahad Medical City, Dabab Street, Sulaimaniya, PO Box 221124, Riyadh 11311, Saudi Arabia; Department of Adult Cardiology, King Fahad Medical City, Dabab Street, Sulaimaniya, PO Box 221124, Riyadh 11311, Saudi Arabia; Department of Adult Cardiology, King Fahad Medical City, Dabab Street, Sulaimaniya, PO Box 221124, Riyadh 11311, Saudi Arabia; Department of Adult Cardiology, King Fahad Medical City, Dabab Street, Sulaimaniya, PO Box 221124, Riyadh 11311, Saudi Arabia; Department of Adult Cardiology, King Fahad Medical City, Dabab Street, Sulaimaniya, PO Box 221124, Riyadh 11311, Saudi Arabia

**Keywords:** Case report, Primary cardiac lymphoma, Acute coronary syndrome, Arrhythmias, Heart failure

## Abstract

**Background:**

Primary cardiac T-cell lymphoma (PCTCL) is an extremely rare subtype of primary cardiac lymphoma. Due to the paucity of clinical data, it poses a challenge from diagnosis to management.

**Case summary:**

A 65-year-old female presented with dyspnoea and epigastric pain. Her electrocardiogram revealed an ST-elevation in leads II, III, aVF, and V4–V6, for which she underwent an emergent coronary angiogram, which revealed normal coronaries. Further multimodality cardiac imaging demonstrated a large right ventricular mass infiltrating the inferior wall of the left ventricle. A percutaneous transvenous biopsy was done, and results confirmed an anaplastic T-cell lymphoma, which prompted the initiation of chemotherapy. Unfortunately, her course became more complicated despite a notable reduction in the mass size on follow-up imaging, as she had an incessant, unstable ventricular tachycardia unresponsive to medical therapy, which led to death within 3 months of presentation.

**Discussion:**

PCTCL has unpredictable presentations. A biopsy is required to reach a definitive diagnosis, alongside multimodality cardiac imaging. Moreover, left ventricular involvement may directly influence survival in PCL and potentially indicate an advanced and fatal stage.

Learning points

- Primary cardiac T-cell lymphoma is a rare condition that can mimic STEMI due to myocardial involvement.
- Multimodality imaging and endomyocardial biopsy are essential to establish the diagnosis.
- Cardiac lymphomas may cause life-threatening ventricular arrhythmias.
- Left ventricular involvement may signify an advanced stage of cardiac lymphoma.

## Introduction

Primary cardiac lymphomas (PCL) constitute a rare entity of cardiac tumours.^1^ The most common subtype is diffuse large B-cell lymphoma.^2^ Another variant is a cardiac lymphoma of T-cell lineage. Yet, clinical data are scarce, since it is extremely rare, with a limited number of published cases globally.^2,3^ PCL responds well to chemotherapy, but the prognosis is generally unfavourable due to a high rate of recurrence.^2^ Notably, left ventricular (LV) involvement is likely to have a direct impact on survival.^4^ We report a ST-elevation myocardial infarction (STEMI)-like presentation of an advanced primary cardiac T-cell lymphoma (PCTCL) infiltrating both the right and the left ventricles, complicated by an incessant ventricular arrhythmia.

## Summary figure

**Figure:**
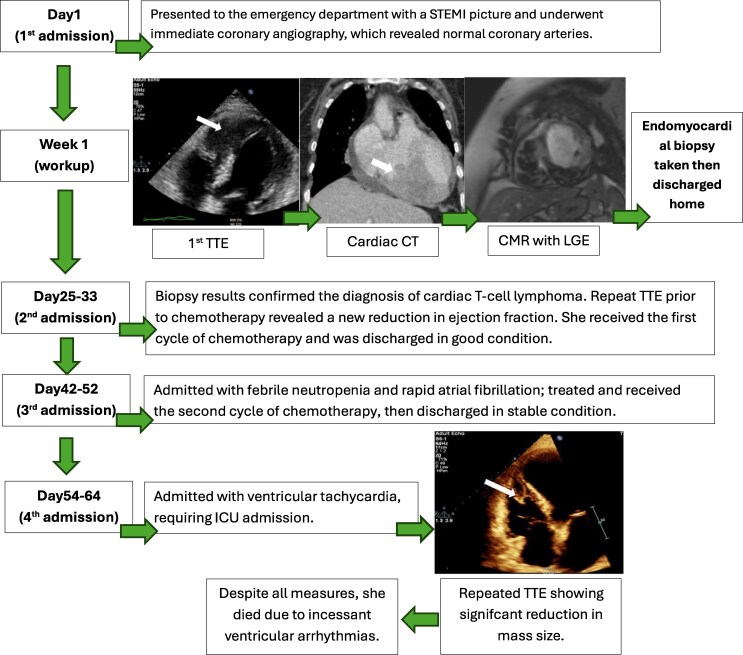


## Case summary

**Figure 1:**
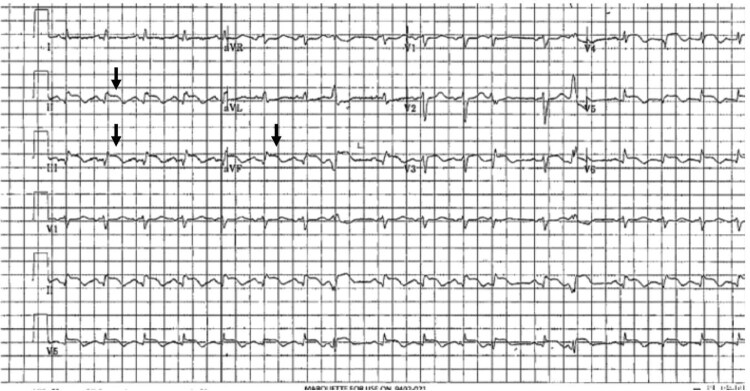
First ECG showing convex ST-elevations in multiple leads (II, III, aVF, and V4–6) mimicking an acute ST-elevation myocardial infarction.

**Figure 2:**
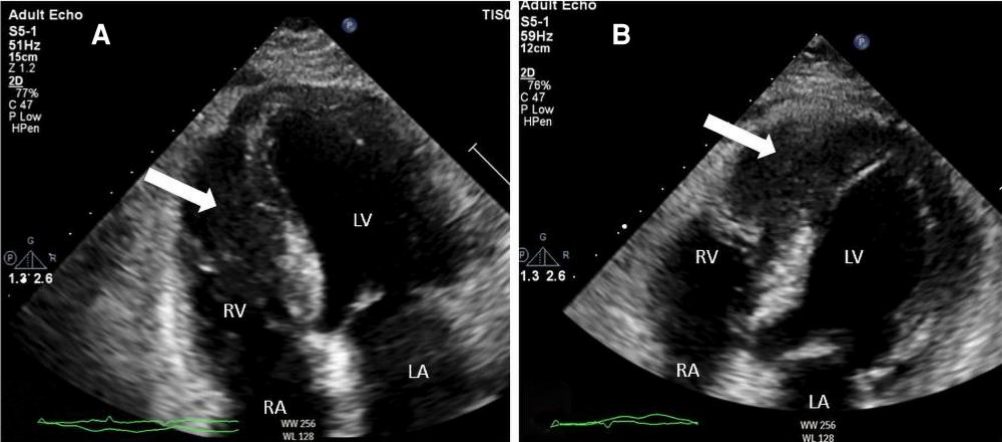
Transthoracic Echocardiography. An apical four-chamber (*A*) and right ventricular focused view (*B*) showing a large homogenous mass (white arrows) measuring 4.8 cm × 2.5 cm in the right ventricular apex and infiltrating the interventricular septum.

**Figure 3:**
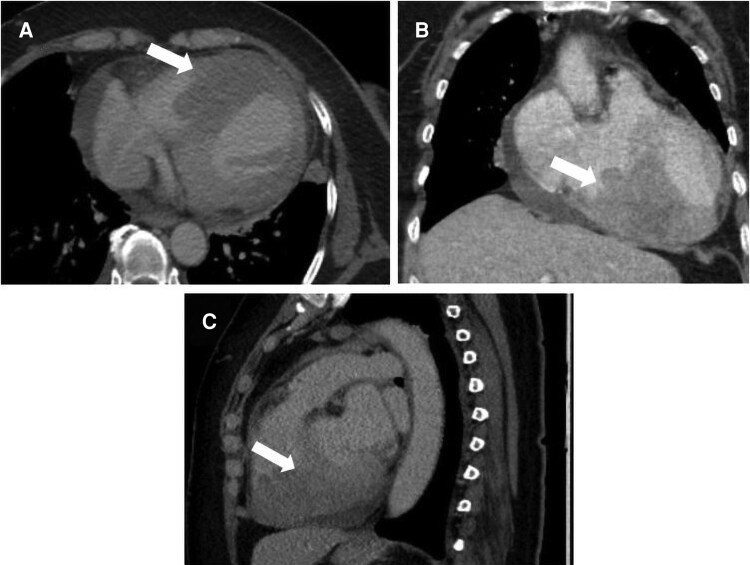
Cardiac computed tomography with contrast showing an enhancing infiltrative soft tissue cardiac mass extending to the right ventricle with involvement of the interventricular septum and the left ventricular inferior walls (*A*, *B*, *C*, white arrows).

**Figure 4:**
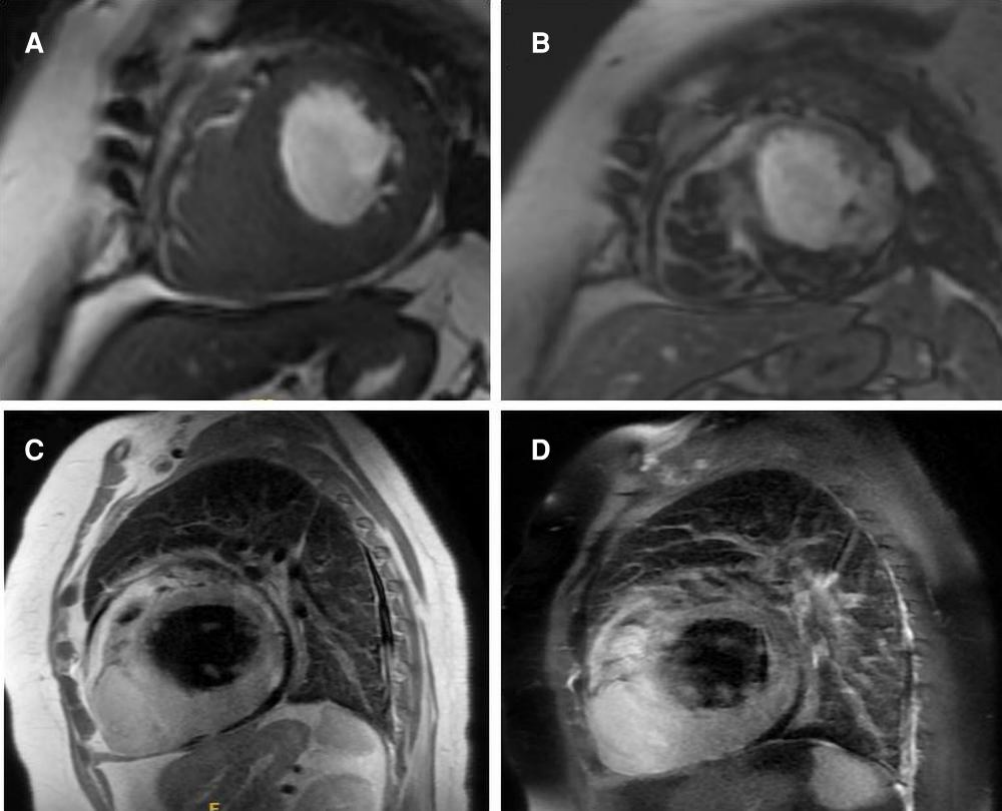
Cardiac magnetic resonance imaging revealed a large infiltrative intracardiac mass, primarily involving the right ventricular apex and infiltrating into the distal interventricular septum and the inferior wall of the left ventricle, as well as part of the adjacent pericardium (*A*). The lesion demonstrates heterogeneous, intense enhancement on LGE sequences, reflecting necrosis, fibrosis, and viable tumour tissue (*B*). On T2-weighted HASTE sequences, the mass appears hyperintense, indicating high water content and cellularity, consistent with a malignant tumour (*C*). On T2-weighted imaging with fat saturation, the mass is isointense to mildly hyperintense relative to myocardium, with suppression of adjacent fat, facilitating clear delineation of the lesion (*D*).

**Figure 5:**
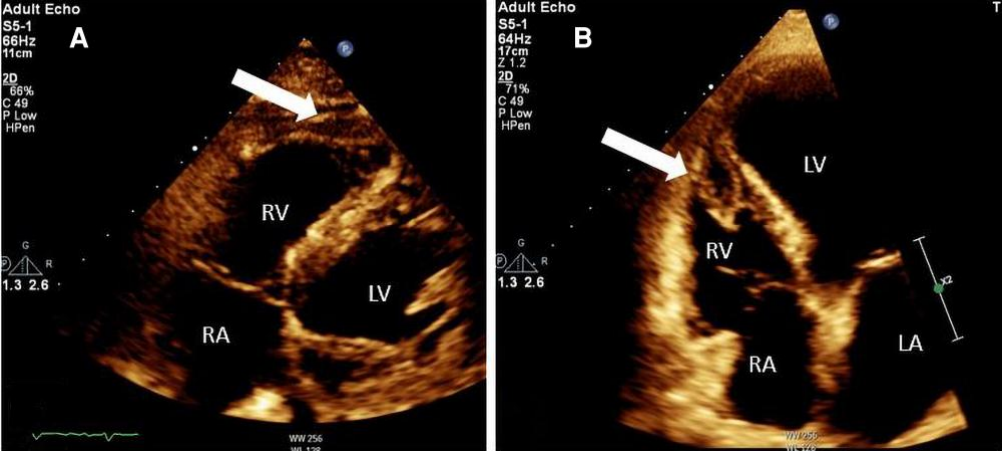
Transthoracic Echocardiography, right ventricular focused view (*A*) and an apical four-chamber (*B*) showing a significant reduction in the mass size (white arrows).

A 65-year-old female with a past medical history of hypertension presented to the emergency department with exertional dyspnoea for 2 months, associated with epigastric pain that was increasing a few hours before presentation. Upon physical examination, she was well-looking and had stable haemodynamics. Vitals were as follows: a blood pressure of 120/80 mmHg, a heart rate (HR) of 80 b.p.m., and peripheral saturation above 95% while breathing ambient air. She had normal first and second heart sounds with no extra sounds. Lungs were clear on auscultation. Her abdomen was soft without tenderness. No peripheral signs of overload. Her ECG revealed an ST-elevation in leads II, III, aVF, and V4–V6 (*Figure 1*). She underwent an emergent coronary angiogram; surprisingly, the coronaries were disease-free, with TIMI III flow. Further history revealed a 2-month history of night sweats, subjective fever, and unintentional weight loss. Examination was negative for lymphadenopathy, hepatomegaly, or splenomegaly. Pertinent laboratory findings included an elevated troponin I 45 ng/L (normal 0–15.6), BNP at 519 pg/ml (normal 0–159), haemoglobin at 11 g/dl (normal 11–16), and leucocyte count 14 × 10⁹/L (normal 3.9–11 × 10⁹/L). Additionally, she tested positive for EBV PCR. HIV screening was negative. Liver, renal, and coagulation profiles were normal. Chest x-ray was significant for cardiomegaly without congestion. She had a multimodality cardiac imaging, initially with a transthoracic echocardiogram (TTE) that revealed a large mass in the right ventricular apex infiltrating the interventricular septum (*Figure 2*). Her ejection fraction was 55%, and there was mild pericardial effusion (see Supplementary material online, *Videos S1* and *S2*). Cardiac contrast-enhanced computed tomography was done, which showed an enhancing infiltrative soft tissue mass (9 cm × 9 cm) extending from the right ventricle and interventricular septum to the left ventricular inferior wall, inseparable from the pericardium (*Figure 3*). A cardiac magnetic resonance imaging (CMR) with late gadolinium sequences demonstrates a heterogeneous, intensely enhanced lesion, reflecting necrosis, fibrosis, and viable tumour tissue. These findings suggest a malignant tumour, such as cardiac lymphoma or sarcoma (*Figure 4*). She then underwent a percutaneous transvenous biopsy from the right ventricular mass; the results confirmed an anaplastic T-cell lymphoma. Immunohistochemistry of the tumour is positive for CD45, CD2, MUM-1, CD4 (focal), CD30, EBER, CD43, CD45RO, and MUM1. Metastatic lesions were ruled out with a computed tomography of the chest, abdomen, and pelvis. During this period, she experienced an episode of supraventricular tachycardia (SVT) that responded to adenosine and then was maintained with 50 mg twice daily metoprolol. A recurrence of the SVT has necessitated adding 200 mg of oral amiodarone twice daily to metoprolol. Chemotherapy was planned with the BV-CHP regimen (brentuximab vedotin, cyclophosphamide, doxorubicin, prednisolone). A repeated TTE before chemotherapy revealed a decrease in her EF to 35%, and heart failure therapy was initiated. The first cycle of chemotherapy was completed within the first month of presentation. She was discharged in good condition. Ten days later, she was hospitalized due to febrile neutropenia associated with atrial fibrillation with rapid ventricular response, which was managed medically and converted to sinus rhythm. After recovery, she received the second cycle of chemotherapy and was again discharged stable. A week later, she was admitted to a local hospital with stable monomorphic ventricular tachycardia (VT) requiring six synchronized cardioversion trials to convert to sinus rhythm. Transferred to our centre for tertiary care, she arrived stable on amiodarone infusion with an HR of 84 b.p.m. The next day, she developed unstable VT at 180 b.p.m., and her blood pressure dropped to 80/40 mmHg. She received three DC shocks, followed by emergent sedation and intubation, and inotropic support. While on lidocaine and amiodarone infusions, she continued to have recurrent slow VTs. Electrolytes were replaced per hospital protocol. A repeated TTE showed a significant reduction in RV mass, but with further LV dysfunction to 25% (*Figure 5*, Supplementary material online, *Videos S3* and *S4*). Unfortunately, despite ongoing treatment for her VT, she arrested, and after 20 min of cardiopulmonary resuscitation, death was declared.

## Discussion

Primary cardiac lymphoma (PCL) is classified as a rare malignant infiltrative cardiac tumour and comprises <2% of cardiac neoplasms.^1^ Petrich *et al*.^4^ and Gordon *et al*.^5^ have published the most extensive retrospective analysis of all published PCL cases. From these two reviews, the most common symptom reported was dyspnoea. Arrhythmias, pericardial effusion, and congestive heart failure are also frequently encountered upon diagnosis. There is a predilection for the right-sided chambers.^4,5^ Left ventricular involvement has been associated with poorer survival outcomes, with a median overall survival of 1 month.^4^ Atrioventricular blocks and atrial arrhythmias are the most common arrhythmias reported, while ventricular arrhythmias were the least observed.^4–6^ Primary cardiac T-cell lymphomas (PCTCL) are described only in a limited number of case reports.^5^ Its occurrence is higher in HIV and those who are immunosuppressed; however, cases have also been documented in immunocompetent individuals.^1^ Presentation is variable depending on the affected site, which can be as simple as a bradyarrhythmia presentation^7^ or challenging presentations, manifesting as STEMI resulting from tumour invasion causing complete coronary occlusion.^3,8^ Diagnosis is established with multimodality imaging and biopsy of the tumour.^2^ Chemotherapy is the cornerstone of management, with up to a 60% response rate and a median of 13 months of progression-free survival.^7^ Despite all the literature published on PCL, we still encounter unusual presentations. Our case was unique since the initial manifestation was a STEMI-like presentation that unmasked an aggressive infiltrating PCTCL. Only a few cases have been reported with ST-elevation secondary to PCL^3,8,9^; to our knowledge, only one was secondary to T-cell lymphoma.^3^ Our case differs from the others described in the literature as the patient’s coronaries were completely normal. The ECG changes seen in our patient may be explained by direct myocardial injury related to the infiltrative process of the PCL.^9^ Moreover, our patient was stable initially and had started two cycles of chemotherapy. The RV mass has significantly reduced in size on her follow-up ECHO; despite that, she developed multiple atrial tachyarrhythmias and, lastly, a persistent fatal VT. It has been observed that arrhythmias were associated with longer survival, possibly due to earlier recognition of the disease.^4^ Yet, our patient deteriorated fast with worsening left ventricular (LV) function. In our view, it was a dual insult to her LV, attributed to the infiltrative nature of the tumour, causing direct muscle injury; in addition, she received doxorubicin, an anthracycline known for its cardiotoxic effects.^10^

In conclusion, primary cardiac T-cell lymphoma can mimic ST-elevation myocardial infarction (STEMI), exhibiting typical ECG changes. Left ventricular infiltration may signify an advanced stage of the disease, which can lead to severe LV dysfunction and life-threatening arrhythmias.

## Lead author biography


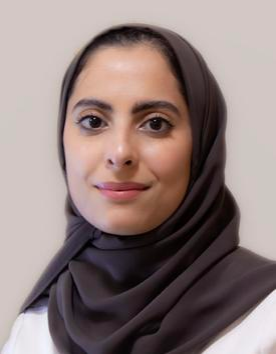


Dr. Alaa Jamal Alobaidli is a Saudi female. She is a passionate cardiology fellow, currently training at King Fahad Medical City in Riyadh. She is interested in advanced cardiac imaging, advanced heart failure, and research.

## Supplementary Material

ytag086_Supplementary_Data

## Data Availability

The data underlying this article are available in the main manuscript or the Supplementary material.
